# Incidence of diabetes mellitus in Spain as results of the nation-wide cohort di@bet.es study

**DOI:** 10.1038/s41598-020-59643-7

**Published:** 2020-02-17

**Authors:** G. Rojo-Martínez, S. Valdés, F. Soriguer, J. Vendrell, I. Urrutia, V. Pérez, E. Ortega, P. Ocón, E. Montanya, E. Menéndez, A. Lago-Sampedro, T. González- Frutos, R. Gomis, A. Goday, S. García-Serrano, E. García-Escobar, J. L. Galán-García, C. Castell, R. Badía-Guillén, G. Aguilera-Venegas, J. Girbés, S. Gaztambide, J. Franch-Nadal, E. Delgado, F. J. Chaves, L. Castaño, A. Calle-Pascual

**Affiliations:** 1 Spanish Biomedical Research Network in Diabetes and Associated Metabolic Disorders (CIBERDEM), Madrid, Spain; 2 Biomedical Research Institute of Malaga (IBIMA), Endocrinology and Nutrition Department, Regional University Hospital of Malaga, Malaga, Spain; 3 Department of Endocrinology and Nutrition, University Hospital Joan XXIII, Pere Virgili Institute (IISPV), Rovira I Virgili University, Tarragona, Spain; 4 Cruces University Hospital, Biocruces Bizkaia Health Research Institute, UPV/EHU, Barakaldo, Spain; 5 Spanish Biomedical Research Network in Rare Diseases (CIBERER), Madrid, Spain; 6 General Laboratory. Regional University Hospital of Malaga, Malaga, Spain; 7 Department of Endocrinology and Nutrition, August Pi i Sunyer Biomedical Research Institute – IDIBAPS, Hospital Clínic of Barcelona, Barcelona, Spain; 8 Spanish Biomedical Research Network in physiopathology of obesity and Nutrition (CIBEROBN), Barcelona, Spain; 9 Bellvitge Biomedical Research Institute (IDIBELL), University of Barcelona, Bellvitge University Hospital, Barcelona, Spain; 10 Department of Endocrinology and Nutrition, Central University Hospital of Asturias/University of Oviedo, Health Research Institute of the Principality of Asturias (ISPA), Oviedo, Spain; 11 Department of Endocrinology and Nutrition, Hospital del Mar, IMIM. Universitat Autònoma, Barcelona, Spain; 12 Department of Applied Mathematics, Malaga University, Malaga, Spain; 13 Department of Health, Public Health Agency of Catalonia, Barcelona, Spain; 14 Diabetes Unit, Hospital Arnau of Vilanova, Valencia, Spain; 15 Department of Endocrinology and Nutrition, Cruces University Hospital, Biocruces Bizkaia Health Research Institute, UPV/EHU, Barakaldo, Spain; 16 EAP Raval Sud, Catalan Institute of Health, GEDAPS Network, Primary Care, Research support unit (IDIAP – Jordi Gol Foundation), Barcelona, Spain; 17 Genomic and Genetic Diagnosis Unit, Research Foundation of Valencia University Clinical Hospital-INCLIVA, Valencia, Spain; 18 Department of Endocrinology and Nutrition, University Hospital S. Carlos of Madrid, Madrid, Spain

**Keywords:** Type 2 diabetes, Epidemiology

## Abstract

Our aim was to determine the incidence of type 2 diabetes mellitus in a nation-wide population based cohort from Spain (di@bet.es study). The target was the Spanish population. In total 5072 people older than 18 years,were randomly selected from all over Spain). Socio-demographic and clinical data, survey on habits (physical activity and food consumption) and weight, height, waist, hip and blood pressure were recorder. A fasting blood draw and an oral glucose tolerance test were performed. Determinations of serum glucose were made. In the follow-up the same variables were collected and HbA1c was determined. A total of 2408 subjects participated in the follow-up. In total, 154 people developed diabetes (6.4% cumulative incidence in 7.5 years of follow-up). The incidence of diabetes adjusted for the structure of age and sex of the Spanish population was 11.6 cases/1000 person-years (IC95% = 11.1–12.1). The incidence of known diabetes was 3.7 cases/1000 person-years (IC95% = 2.8–4.6). The main risk factors for developing diabetes were the presence of prediabetes in cross-sectional study, age, male sex, obesity, central obesity, increase in weight, and family history of diabetes. This work provides data about population-based incidence rates of diabetes and associated risk factors in a nation-wide cohort of Spanish population.

## Introduction

Type 2 diabetes mellitus is one of the most serious health problems of our time. Data from numerous studies agree on the continuous growth of its incidence and prevalence rates throughout the world, with unacceptable human, social, and economic costs. Diabetes has become one of the main causes of cardiovascular disease, blindness, non-traumatic amputations of the lower limbs, kidney failure, and death throughout the world^1^. In addition, its association with the presence of cancer has recently been demonstrated^2^. Therefore, diabetes is a major challenge to public health in all countries. Understanding its epidemiology becomes supremely important for both determining the health status of the population and planning resources for their care, early diagnosis, and prevention.

The increase in the prevalence of diabetes in the world is multifactorial, and can be partly attributed to population aging, higher survival of people with diabetes due to improved medical care, and changes in lifestyle linked to increased urbanisation with sedentary behaviour and unhealthy eating profiles leading to increased obesity^1^. The impact of changes in lifestyle is greater in low income countries^3^.

A substantial proportion of people with diabetes are undiagnosed. In Europe, this group represents 37.9% of total diabetes, which, although one of the lowest in the world, means that 22 million people are at an increased risk of developing cardiovascular diseases^1^. In Spain, the di@bet.es study found that nearly half of the detected cases were undiagnosed diabetes^4^.

Most nationwide European studies on diabetes incidence are based on health service registries, and have reported incidences between 3 and 6 cases/1000 person-years^5,6^. However, as far as we know, there are no nation-wide population-based incidence studies on diabetes diagnosed by oral glucose tolerance test (OGTT).

Type 2 diabetes is a potentially preventable disease^7,8^, and the studies on its incidence have led to examination of the variables associated with a greater risk for developing this disease. Type 2 diabetes is a polygenic disease very influenced by environmental factors and, therefore, it is necessary to perform specific population-based incidence studies, as recommended by the International Diabetes Federation (IDF)^9^. Of special interest are the studies at national level, which include results about the differences among natural populations and are necessary for national health systems to implement screening and prevention programs as basic tools to prevent the epidemic.

The di@bet.es study was the first national study in Spain designed with the objective of determining the prevalence and incidence of diabetes by OGTT. The results of the cross-sectional study were published in 2012^4^. The objective of the present study was to describe the incidence of diabetes in Spain through the re-evaluation of this cohort, as well as to examine the main risk factors related to development of diabetes.

## Results

The main characteristics in the cross-sectional study of the subjects included and non-included in the incidence study are shown in Table 1. Men participated less than women and the re-evaluated subjects were one year older and had a slightly higher fasting glucose (0.06 mmol/L) than the non-participants. Participants presented more frequently a family history of diabetes, more adherence to the Mediterranean diet, and lower frequency of current smoking (more ex-smokers in this group). The rest of the variables studied were similar in both groups.

**Table 1:** Main characteristics of the population in the cross-sectional study. Comparison between participants and non-participants in the follow-up.

	Non- participants	Participants	p^a^
n	1189	2408	
Sex (%male)	43.7	39.7	0.01
Age (years)	46.7 ± 16.5	47.9 ± 14.7	0.03
BMI (kg/m^2^)	27.6 ± 5.2	27.5 ± 4.7	0.7
Systolic blood pressure (mmHg)	128.6 ± 19.2	128.7 ± 18.5	0.8
Dyastolic blood pressure (mmHg)	76.2 ± 12.6	76.3 ± 10.4	0.7
Fasting glucose (mmol/L)	5.04 ± 0.7	5.1 ± 0.7	0.008
Post OGTT Glucose (mmol/L)	5.8 ± 1.7	5.7 ± 1.7	0.4
Waist (cm)	92.4 ± 14.2	92.2 ± 13.1	0.6
Family history of diabetes (%)			
No	54.8	49.8	0.009
Yes, one relative	25.5	26.7	
Yes, two or more relatives	19.7	23.5	
Physical activity (%)			
Low	41.1	42.3	0.7
Moderate	34.6	33.8	
High	24.2	23.8	
Mediterranean diet score (14p)			
<8p	68.4	64.4	0.02
> = 8p	31.6	35.6	
Smoking (%)			
Never	49.0	49.8	0.001
Former smoker	19.1	24.4	
Current smoker less than 15 cig/day	15.5	14.5	
Current smoker more than 15 cig/day	16.4	11.4	
Dyslipidaemia (%)	25.7	26.6	0.5
Obesity (%)	28.1	26.2	0.1
Central obesity (%)	66.0	70.0	0.004
High blood pressure (%)	37.1	38.8	0.4
OGTT result (%)			
Normal	86.6	87.8	0.3
IFG	5.3	4.2	
IGT	6.1	6.4	
IFG + IGT	2.0	1.5	
Education level (%)			
None	10.1	8.4	0.1
Basic	49.2	47.4	
High school	23.9	23.6	
College	16.8	17.6	

^a^p = signification level of t-student, Mann-Whitney or chi2 test according to type of variable.

In total, 156 people developed diabetes over the 7.5 year follow-up, which yields a 6.5% cumulative incidence. The raw incidence rate in the sample was 8.5 per 1000 person-years (IC95% = 7.3–10.1) (Table 2).

**Table 2:** Incidence of diabetes according to the main exposure factors present in the cross-sectional study.

	N° at risk	n° developing diabetes	person/years	Incidence rate per 1000 person-years (95% CI)	OR^a^ (95% CI)	OR^b^ (95% CI)
All sample	2408	156	18088	8.6 (7.3–10.1)	—	—
Sex						
Women	1451	81	10883	7.4 (5.9–9.3)	Ref. cat.	Ref. cat.
Men	957	75	7205	10.4 (8.2–13)	1.5 (1.1–2.1)	2.7 (1.6–4.5)
OGTT result						
Normoglycemia	2115	75	15892	4.7 (3.7–5.9)	Ref. cat.	Ref. cat.
Isolated IGT	154	35	1156	30.3 (21.1–42.1)	10.8 (5.7–20.2)	7.9 (4–15.5)
Isolated IFG	102	27	761	35.5 (23.4–51.6)	14.4 (7.5–27.6)	11.7 (5.9–23.3)
Combined IFG-IGT	37	19	278	68.3 (41.1–106.6)	42.3 (16.3–109.5)	48.8 (17.1–139.8)
P for trend					<0.0001	<0.0001
Obesity						
BMI < 25 kg/m^2^	749	15	5628	2.7 (1.5–4.4)	Ref. cat.	Ref. cat.
BMI 25–30 kg/m^2^	1018	57	7662	7.4 (5.6–9.6)	1.9 (0.9–3.9)	1.2 (0.6–2.3)
BMI ≥ 30 kg/m^2^	626	82	4691	17.5 (13.9–21.7)	4.6 (2.3–9.2)	2.3 (1.1–4.6)
P for trend					<0.0001	<0.0001
Central obesity (Waist ≥94 cm in men and ≥80 cm in women)						
No	718	8	5400	1.5 (0.6–2.9)	Ref. cat.	Ref. cat.
Yes	1677	146	12596	11.6 (9.8–13.6)	10.6 (2.6–43.5)	3.4 (1.5–7.8)
Family History of diabetes (first-degree relatives)						
No	1569	73	11794	6.2 (4.9–7.8)	Ref. cat.	Ref. cat.
Yes	839	83	6294	13.2 (10.5–16.3)	2.4 (1.5–3.9)	2.3 (1.6–3.3)

^a^ORs were calculated for each variable by logistic regression adjusted for age and sex (sex was adjusted for age).

^b^ORs were calculated using a single logistic regression model (all variables listed are introduced in the analysis at once) and additionally adjusted for presence of high blood pressure, level of physical activity (IPAQ), education level, and Mediterranean diet score. Ref. cat. = Reference category.

The estimated incidence of diabetes adjusted for the age and sex structure of the Spanish population and the form of detection of diabetes was of 11.6 cases/1000 person-years (IC95% = 11.1–12.1). The incidence of known diabetes was 3.7 cases/1000 person-years (IC95% = 2.8–4.6) and, therefore, the incidence of unknown diabetes was 7.9 cases/1000 person-years (IC95% = 5.3–8.1).

**Figure 1:**
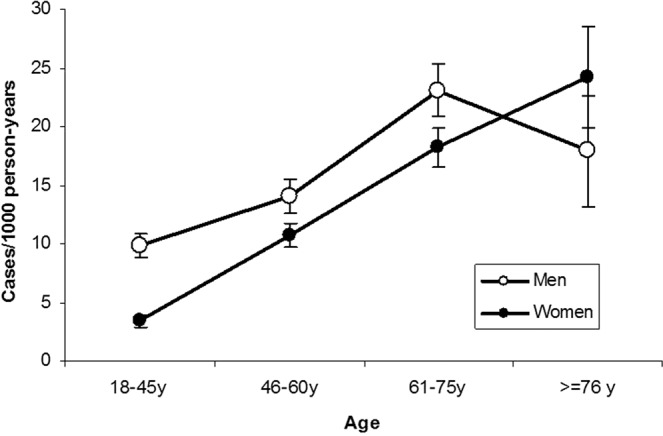
Incidence of diabetes according to sex and age, adjusted for diagnostic method.

As expected, the incidence of diabetes increased with age and was higher among men (13.4 cases/1000 person-years IC95% = 12.6–14.2) than among women (9.9 cases/1000 person-years 95% CI = 9.3–10.4) adjusted for the form of detection of diabetes. Nevertheless, among people older than 75 years there was no difference between sexes (Fig. 1).

The OR of developing diabetes was nearly 3-fold higher among men that among women (OR = 2.7 (1.6–4.5)), adjusted for all the other variables studied (Table 2).

Both impaired fasting glucose (IFG) and impaired glucose tolerance (IGT), isolated or in combination, were risk factors associated with diabetes. There was a significant risk gradient IGT < IFG < IGT + IFG (P for trend < 0.0001) in addition to a significant interaction with sex (P = 0.02).

The presence of obesity and central obesity were related to the risk of diabetes, but overweight (BMI between 25 and 30 Kg/m^2^) did not reach significance; although there was a gradient in risk, as shown by the P for trend significant (Table 2). Family history of diabetes also contributed to the risk, doubling the incidence. High blood pressure, physical activity, the score of adherence to the Mediterranean diet, and dyslipidemia yielded not significant associations neither in the monovariant nor in the adjusted models (data not shown).

Since there was an interaction between the presence of dysglycemia (IFG and/or IGT) and sex in the explanation of the risk of diabetes (p < 0.0001), we performed a stratified analysis separating each sex to reveal the interaction. The incidence of diabetes in normoglycemic men (7.2 cases/1000 person-years, 95% CI = 5.4–9.6) was significantly higher than in women (3.1cases/1000 person-years, 95% CI = 2.2–4.5), but in presence of dysglycemia, both incidence (32.1cases/1000 person-years, 95% CI = 21.7–45.8 in men vs 40.4 cases/1000 person-years, 95% CI = 30.1–53.2 in women) and adjusted OR (3.5 (1.9–6.2) in men vs 12.4 (7.1–21.8) in women, were higher in women than in men.

## Discussion

The incidence of diabetes was determined by means of a representative sample of the Spanish population and using the best available diagnostic methods (OGTT and/or HbA1c). The overall incidence adjusted for the Spanish population, and calculated taking into account that not all subjects underwent all diagnostic tests, was of 11.6 cases/1000 person-years. The incidence of known diabetes has only been a fraction of the total diabetes detected: 3.71 cases/1000 person-years, a figure that can be assimilated to the incidence of diabetes diagnosed in the national health system.

In Spain, current data about incidence of diabetes in the general population are scarce and partial. In the Lejona study, performed in the north of Spain in 1995, the incidence of diabetes was of 8.2 cases/1000 person-years 10-year prospective study on the incidence^10^. In 2008, the revaluations of two local studies were completed, one in the north (Asturias Study)^11^ and another one in the south (Pizarra study)^12^ of Spain. In both of them, approximately 700 subjects were followed for an average of 6–7 years. Both studies were conducted with a very similar methodology, but the incidence in the north was of 10.8 cases/1,000 person-years and in the south of 19.1 cases/1,000 person-years. One possible reason for the apparent disparity in the results of these two studies, practically contemporaneous, could be the higher prevalence and incidence of obesity in the population of Pizarra, where the mean weight gain during the follow-up doubled the one of the Asturias study^11,12^. The results that we present from the di@bet.es cohort are concordant with the studies carried out in the north, but lower than those carried out in the south of Spain. In our cohort, the diabetes prevalence has been previously shown to be higher in the south of Spain compared to the other parts of the country^11^.

In 2013, data on the incidence of diabetes from the Spanish participants in the EPIC cohort, made up of voluntary donors from several Spanish localities followed for 12 years, were published^13^. Adjusted rates varied between 4.2 and 7.2 cases/1000persons-year in men and 3.5 and 4.3 cases/1,000 person-years in women, with abdominal obesity being one of the main risk factors. These lower incidence rates with respect to population-based studies may be due to the sample selection, since the EPIC comprised healthy volunteers (blood donors and workers), while in the other Spanish studies, including the present results, sampling was composed of general population, not necessarily healthy. The diagnosis of diabetes by OGTT also increases the number of incident cases. The wide population studies that use this diagnostic tool are very scarce. Something similar happens with the study based on the working population recently published in Spain “Incidence of Diabetes in the Working Population in Spain”^14^: the incidence (5.0 cases/1000 person-years) was very low and similar to the one found in EPIC and other studies in which the working population was mainly involved.

There are no studies on the incidence of diabetes in Europe as a whole, and only few countries have data at a national level, usually based on health records. The study by Norhammar *et al*.^5^ in Sweden, with an overall incidence of 4.2 per 1000 person-years, or the study by Holst^6^ in Denmark, with incidences of 6.2 in men and 4.4 in women, are examples of this, but their findings are clearly lower than those of our study. The IDF estimates that the prevalence of diabetes in Europe will increase by 16% between 2017 and 2045^9^. This estimate is equivalent to an incidence rate of 4.2 cases/1000 person-years, similar to the one calculated for diagnosed diabetes by health systems that do not usually use OGTT as a diagnostic method. The incidence of diabetes in these works is similar to the incidence of previously known diabetes in our study (3.7 cases/1000 person-years).

The studies conducted in Europe (mostly local) in the last years, based on glucose determinations and rarely using OGTT, reveal the heterogeneity of diabetes incidence among the different studies^14–22^ ranging from 5 per 1000 cases of the ICARIA study^14^ in working population, to more than 17 per 1000 of the Hoorn study^18^ with OGTT in a population with mean age around 60 years old. These differences may be due to (besides the methodology) the important differences between countries in the prevalence of obesity, physical activity, or eating patterns, which might partially explain the variation in diabetes prevalence^23^. On the other hand, our incidence rates are clearly lower than those reported for high risk populations, like the North-american^19,24^.

In the di@bet.es study, the variables associated with the presence of diabetes in the cross-sectional study were age, sex, educational level, obesity, abdominal obesity, hypertension, low levels of HDL cholesterol, and high levels of triglycerides as well as the family history of diabetes^4^. In the current work, most of these variables are also associated with incident diabetes, except for hypertension and dyslipidemia. This may be due to the fact that these variables are not causative of diabetes, but they rather share a common pathophysiology, probably linked to the loss of insulin sensitivity^25^. The main predictor variable has been the presence of dysglicemia in the baseline study, as it happened in other studies^14,17,18,26^. The presence of IGT conferred a lower risk than IFG, while the combination of both multiplied the risk of developing diabetes, especially in women. A similar effect has been found in the ADDITION-Denmark study^26^. The importance of detecting an early dysglicemia lies not only in the increased risk of developing diabetes over time, but also in the higher risk of developing cardiovascular and renal disease, and the higher mortality than normoglycemic subjects^27^. In our work, the risk associated with the presence of IGT or IFG is somewhat higher than the one described in the meta-analysis by Gerstein^28^.

Important differences have been found in the prevalence and incidence of diabetes according to sex^3^. In women, the incidence rates were lower than in men, but only in normoglycemic women. However, the presence of dysglycemia increased much more the risk in women that became statistically equal to men, and resulted in a significantly higher OR (significant interaction disglycemia x sex), which, as far as we know, has not been described in other studies.

The ability of glucose post-OGTT to predict diabetes, together with its relationship with mortality and cardiovascular risk^29^, makes it one of the main risk factors to be taken into account, although the complexity of its measurement makes it an invalid test for screening. In our study, glucose post OGTT predicted the risk of diabetes incidence, and, as already mentioned, its simultaneous alteration with basal glycemia multiplied the risk.

Overall and central obesity were independently associated with diabetes. It has been shown^30^ that, from a certain age, obesity is associated with a reduction in the years of life free of diabetes and an increase in the years lived with diabetes.

The adjustment of the models with the family history of diabetes, the presence of high blood pressure, the adherence to the Mediterranean diet, or the intensity of physical exercise hardly modify the magnitude of the OR of the models adjusted only for age and sex. In our study, we did not find a direct effect of adherence to the Mediterranean diet and the level of physical activity on the incidence of diabetes. This may possibly be due to the limited sample size or the lack of precision of some variables as synthetic as the ones used.

The strengths of this study are that the sample has been chosen randomly from all over Spain, with people aged 18 and over, most of whom underwent an OGTT to diagnose diabetes. In the follow-up, HbA1c was also used, which guarantees the capture of most of the incident diabetes. Most published studies on diabetes incidence perform incidence estimates based on health records or self-reported diabetes and therefore may underestimate the real incidence of diabetes^5,6,26,31,32^.

Some limitations deserve mention. Our sample is composed of white people and, therefore, it cannot be generalised to other ethnic groups. The participation in the follow-up was of 66%, however, we have found few differences between people who participated in the follow-up and those who did not, and as a result, the possible participation bias has been minimal. Moreover, diabetes incidence in Spain may not be representative of other countries due to different dietary habits, physical exercise, or genetic background, although these variables have much less influence on the risk of diabetes than the presence of obesity or previous dysglycemia.

Spain has one of the highest life expectancies in the world (only less than Japan and Switzerland) and one of the lowest cardiovascular disease mortality rates in Europe, despite the high rates of metabolic diseases (obesity, diabetes, or hypertension) found in this and other studies^33^. This apparent paradox can be explained by the interaction between the genetic variants selected over centuries in the Mediterranean area with changes in current lifestyles (abandonment of the Mediterranean diet and sedentary lifestyle)^34^, issues that need additional research to be answered.

In conclusion, this work provides data about population-based rates of diabetes incidence and associated risk factors in a nation-wide cohort from Spanish population.

## Methods

### Study design, setting and population

The di@bet.es epidemiological trial is a population based cohort study. The initial cross-sectional study was undertaken between 2008 and 2010 using a random cluster sampling of Spanish population^4^. The sample size was calculated assuming a diabetes prevalence of 15% of the population, with an error lower than 1%. The di@bet.es study sample consisted of 5072 subjects older than 18 years, randomly selected from the National Health System registries distributed into 100 clusters (primary health care centres). Exclusion criteria in the cross-sectional study were: serious illness, pregnancy, recent delivery or lactation, surgery within the previous month, or any other disabling situation that prevented participation.

**Figure 2:**
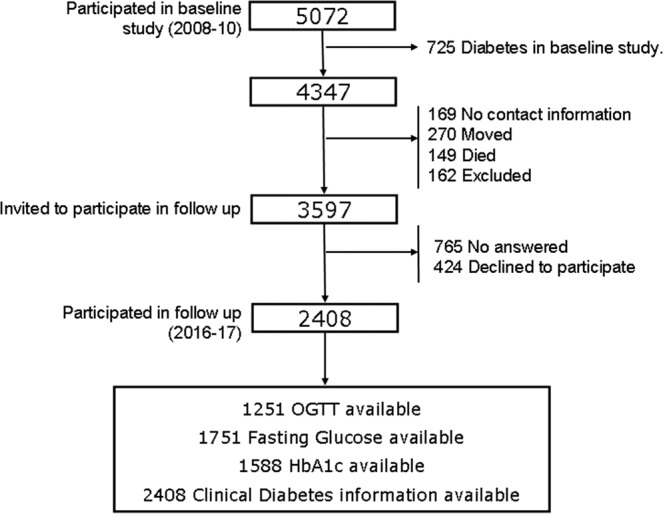
Participation flow chart.

The cohort was re-evaluated in 2016–17 (follow-up time was 7.5 ± 0.6 years). All subjects who had completed the baseline study (n = 5072) were invited by letter and by phone to attend another clinical examination. The 725 subjects who had diabetes at baseline were excluded from all the incidence calculations. Thus, the at-risk sample included 4347 people. As with the cross-sectional study, people with serious illness, pregnancy, recent delivery or lactation, or surgery within the previous month were excluded. Finally, 2408 subjects completed the follow-up (Fig. 2**)**.

The research was carried out in accordance with the Declaration of Helsinki (2008) of the Word Medical Association. Both the cross-sectional study and the follow-up study were approved by the Ethics and Clinical Research Committee of the Hospital Regional Universitario de Malaga (Malaga, Spain). All the participants were informed about the nature of the study and provided written informed consent in the two phases of the study, document approved by the committee mentioned above.

### Procedures

The participants were invited to attend a single examination visit at their health centre with a nurse specially trained for this project. Information was collected using an interviewer-administered structured questionnaire, followed by a physical examination, blood sampling, and an oral glucose tolerance test (OGTT).

A structured questionnaire included closed questions was used to collect the following data: sex, age, instruction level (none, basic, high school or college), personal history of diabetes (yes/no), high blood pressure (yes/no) and dyslipidemia (yes/no), family history of diabetes (yes/no), medications, specifically asking about diabetes, high blood pressure or dyslipidemia medication; frequency of food consumption, and physical activity.

The physical examination included measurement of body weight, height, and waist and hip circumferences that were performed using standardised methods^35^. Besides, body mass index (BMI = weight/height^2^), and waist to hip ratio (WHR) were calculated. Blood pressure was measured with the subject seated after 5 minutes of rest and 2 minutes after the first measurement. A third measure was performed if there was a difference greater than 10% between the first two measurements.

Participants with baseline capillary blood glucose levels lower than 7.8 mmol/L (measured by OneTouch® system, Lifescan, Johnson & Johnson, S.A., Madrid), and not receiving treatment for diabetes, underwent a standard oral glucose tolerance test (OGTT) with 75 g of glucose dissolved in 200 ml of water. In this way, fasting and 2 h venous samples were obtained (8 to 10 hours fasting samples were obtained between 8:30am and 10:00 am in gel tubes suitable with a vacuum blood extraction system), the samples were allowed to stand for 30′. After centrifugation, the serum was separated from the clot and frozen (in cross-sectional study) or refrigerated (in follow up). All samples were analyzed in the same central laboratory after shipment through a specialized courier (Cerba International Laboratory in Barcelona) in the cross-sectional study, and the General Laboratory of the Hospital Regional Universitario of Malaga in the follow-up). In both stages, glucose was determined by the hexokinase enzymatic method, total cholesterol by cholesterol oxidase enzymatic method, HDL cholesterol by direct method, triglycerides by glycerol phosphate oxidase enzymatic method, LDL cholesterol was calculated by Friedewald formula. HbA1C by high-performance liquid chromatography (analyzer ADAMS A1C HA-8180V, ARKRAY^R^) were measured only in follow up study.

The samples were deposited in the Biobank of the Hospital Regional Universitario de Malaga-IBIMA, which belongs to the Andalusian Public Health System Biobank, and the biorepository CIBERDEM managed by IDIBAPS Biobank (Barcelona, Spain).

In follow up, people who did not want to fully participate in the complete study were asked to take a short survey in order to collect information about the drugs they were taking (to determine the existence of clinical diabetes, hypertension or dyslipidemia in treatment), if they were on some type of diet, and self-reported weight.

### Assessment of outcomes in follow up

Incident diabetes was defined as fasting serum glucose equal or higher than 7 mmol/L or 2 hour post load serum glucose equal or higher than 11 mmol/L or HbA1c equal or higher than 6.5% (47.54 mmol/molHb) or use of glucose-lowering medication at the follow-up examination^36^. “Known diabetes” was established when the subject reported having diabetes and/or being treated with diabetes drugs.

### Main risk/exposure factors

The following variables from cross-sectional study were considered: first, glucose impairment category in the cross-sectional study: subjects were classified according to their blood glucose as having a normal OGTT or impaired fasting glucose (IFG) or impaired glucose tolerance (IGT), or both following Alberti and Zimmet^37^. Second, obesity (BMI equal or higher than 30 kg/m^2^) or overweight (BMI between 25 and 30 kg/m^2^). Third, central obesity presence defined as waist circumference >94 cm in men or >80 cm in women^38^.

### Secondary exposure factors and potential confounders

The following variables from cross-sectional study were recorded: age, educational level (none, basic, high school or college), physical activity by the International physical activity questionnaire (IPAQ)^39^, smoking (current, ex- or never smoker), high blood pressure (blood pressure equal or higher 140/90 mmHg or receiving antihypertensive treatment), dyslipidemia (triglycerides were equal or higher 1.7 mmol/L or HDL cholesterol less than 1.03 mmol/L in men or less than 1.29 mmol/L in women or medication), and family history of diabetes (at least one first degree relative with diabetes). A qualitative food frequency questionnaire was administered face-to-face by a trained dietitian^40^. Annual frequency consumption of 50 food items was specified in 11 different categories as follows: never/seldom, 1 and 2–3 times/month, 1, 2–3 and 4–6 times/week and 1, 2, 3, 4 and >4 times/day, and a 14-point Mediterranean diet score was calculated.

### Statistical analysis

Data are presented as means ± SD, or proportions. Differences in baseline variables according to participation in follow-up were determined by the *t*-test for independent samples, the Mann-Whitney test, or chi^2^ test when appropriate. The sample incidence rates (IRs) were calculated as number of events/person-time at risk for diabetes, assuming a constant incidence over time. The estimation of the population incidence rate was calculated taking into account that not all the participants were diagnosed with the four possible criteria (previous diabetes diagnosed after the cross-sectional study, fasting glucose, postload glucose or HbA1c) and adjusted for sex and age by direct method using as reference the Spanish population (https://www.ine.es/, accessed June 2009).

Multivariate analysis was performed using logistical regression for odd ratios (ORs) calculation adjusted for potential confounders. The test of Hosmer and Lemeshow was used to check the goodness of fit. For IRs and ORs, 95% confidence intervals were computed. Analyses were made using SPSS v20 (IBM., Chicago, IL, USA).

## Data Availability

The datasets generated during and/or analyzed during the current study are available from the corresponding author on reasonable request.
